# Raman Spectroscopy *vs* Quantitative Polymerase Chain Reaction In Early Stage Huanglongbing Diagnostics

**DOI:** 10.1038/s41598-020-67148-6

**Published:** 2020-06-22

**Authors:** Lee Sanchez, Shankar Pant, Kranthi Mandadi, Dmitry Kurouski

**Affiliations:** 1 Department of Biochemistry and Biophysics, Texas A&M University, College Station, Texas 77843 United States; 2 Texas A&M AgriLife Research and Extension Center at Weslaco, Texas, 78596 United States; 3 Department of Plant Pathology and Microbiology, Texas A&M University, College Station, Texas 77843 United States; 4 The Institute for Quantum Science and Engineering, Texas A&M University, College Station, Texas 77843 United States; 5 Present Address: Agricultural Research Service, U.S. Department of Agriculture, Stillwater, OK United States

**Keywords:** Applied optics, Optical physics, Optical techniques, Plant stress responses, Abiotic, Biotic

## Abstract

Raman spectroscopy (RS) is an emerging analytical technique that can be used to develop and deploy precision agriculture. RS allows for confirmatory diagnostic of biotic and abiotic stresses on plants. Specifically, RS can be used for Huanglongbing (HLB) diagnostics on both orange and grapefruit trees, as well as detection and identification of various fungal and viral diseases. The questions that remain to be answered is how early can RS detect and identify the disease and whether RS is more sensitive than qPCR, the “golden standard” in pathogen diagnostics? Using RS and HLB as case study, we monitored healthy (qPCR-negative) in-field grown citrus trees and compared their spectra to the spectra collected from healthy orange and grapefruit trees grown in a greenhouse with restricted insect access and confirmed as HLB free by qPCR. Our result indicated that RS was capable of early prediction of HLB and that nearly all in-field qPCR-negative plants were infected by the disease. Using advanced multivariate statistical analysis, we also showed that qPCR-negative plants exhibited HLB-specific spectral characteristics that can be distinguished from unrelated nutrition deficit characteristics. These results demonstrate that RS is capable of much more sensitive diagnostics of HLB compared to qPCR.

## Introduction

Plant diseases can be caused by a variety of different pathogens, such as viruses, fungi and bacteria. Huanglongbing (HLB), or citrus greening, is a devastating disease that plagues citrus trees in Asia, Africa, and more recently the Americas^1^. In the United States, HLB is associated with *Candidatus* Liberibacter asiaticus (*C*Las), a gram-negative bacterium that inhabits the plant phloem in uneven and variable titers^2,3^. Limited knowledge is available on *C*Las bacterium due to our inability to culture it in the laboratory. The bacterium is transmitted by the highly mobile Asian citrus psyllid (*Diaphorina citri*) enabling proliferation of HLB on large agricultural areas. The disease is also able to spread by grafting.

In late stages, HLB-infected plants have asymmetric mottling on the leaves and lopsided and green fruits. HLB causes fruit drop and poor fruit quality, e.g. smaller size, less juice yield, higher acidity and lower sugar content. Early stages of the disease, however, have minimal to no symptoms and the bacteria can remain undetectable even by quantitative polymerase chain reaction (qPCR) in the trees for several years after initial infection^4^. This, wherein, lies a significant challenge in regard to HLB management. Farmers are reluctant to remove trees that have normal, healthy appearances and keep producing fruits. However, if such trees have *C*Las bacteria, they allow for psyllids to quickly spread this devastating disease over large distances. Therefore, it becomes critical to detect HLB at the early (pre-symptomatic) stage and develop appropriate intervention strategies for HLB containment.

In disease diagnostics, the most predominant methods used to confirm a pathogen are PCR and/or enzyme-linked immunosorbent assay (ELISA). Key limitations with these techniques are that they are not portable and there are issues with sensitivity and specificity^5,6^. Variations to the ELISA and PCR methods such as LAMP-PCR^7,8^, or lateral-flow strip assays^9,10^, which to some extent are portable for in-field diagnostics, still face similar issues of sensitivity and specificity^5,6^. Currently, quantitative real time PCR (qPCR) is the most widely employed technique for detection and quantification of the HLB in citrus^11,12^. However, these assays are not high throughput nor is it cost effective to frequently analyze every tree in a field, as would be ideally needed for effective diagnostics and monitoring of HLB^12–14^.

These limitations have motivated the search for alternative approaches for effective HLB diagnostics. Sankaran and co-workers proposed to use visible-near infrared reflectance spectroscopy coupled to multivariate statistical analysis to detect HLB^15^. Although the reported approach showed high accuracy in HLB diagnostics, it was not specific, as the separation could be attributed to color change associated with nutritional stress or other plant stressors and not specifically with HLB stress^15^. Sankaran and co-workers also explored the use of mid-infrared spectroscopy (mid-IR) for HLB diagnostics^16^. Unlike visible-near infrared reflectance, mid-IR is able to probe changes in a plant’s chemical composition that is caused by a pathogen or other stressor. Between healthy, HLB infected, and nutrient deficient groups, they were able to achieve a class separation accuracy of 87.9%^16^. Despite the effectiveness of this method to distinguish and separate classes, the procedures involved in this study required that the plant samples be ground and dried in order to minimize the contribution that water gives to the spectral readings. Raman spectroscopy (RS) is a spectral approach that allows for non-destructive, diagnostic measurements to be taken on citrus leaves in-field^17^. We recently demonstrated that a hand-held Raman system in combination with chemometric analyses could be used to distinguish between healthy, HLB (early and late stage based on symptoms) infected citrus trees, as well as plants suffering from nutrient deficits (ND)^18^. The detection rates of Raman-based diagnostics of healthy vs HLB infected vs nutritional were ~98% for grapefruit and ~87% for orange trees, whereas the accuracy of early vs late stage HLB infected was ~85% for grapefruits and ~94% for oranges. We also demonstrated that RS can be used for diagnostics of fungal diseases on wheat, sorghum and corn^19,20^, as well as viral rose rosette disease on roses^21^.

## Results and Discussion

In our previous study, we used ‘healthy’ orange and grapefruit plants that were grown in the field within close proximity to HLB infected trees as a control for diagnostics of HLB infection^18^. These ‘healthy’ trees exhibited no symptoms associated with HLB and were negative to this pathogen by qPCR. However, it is known that once HLB appears in an area, it can rapidly spread to nearby citrus plants via insect vectors. At the same time, it can take several months or years before the bacteria accumulate to levels detectable by conventional PCR diagnostics. Therefore, it is possible that such ‘healthy’ qPCR-negative plants could already contain bacteria that were vectored by psyllids. This observation raises two questions: 1) what is the true HLB status of the in-field ‘healthy’ (IFH) trees that are surrounded by HLB trees, and 2) is RS sensitive enough to determine the signatures of early HLB-associated spectral changes, before detection by qPCR?

**Figure 1:**
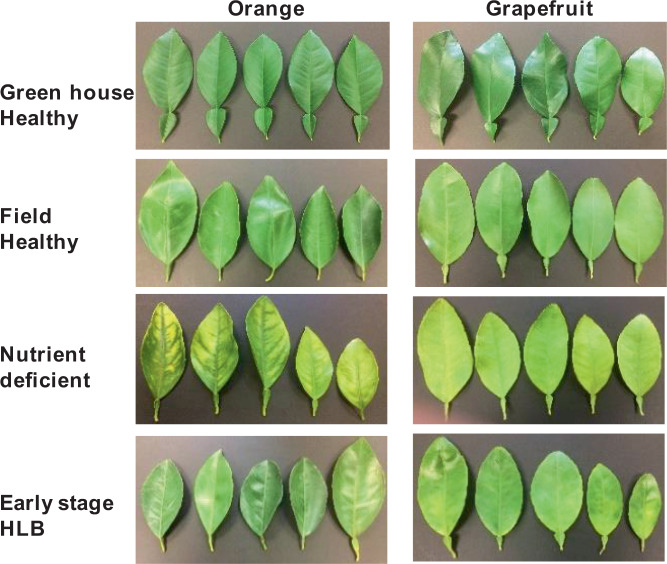
Leaf samples collected from greenhouse healthy (GHH) and field healthy leaves (IFH), as well as leaves from both orange and grapefruit trees with nutrient deficit (ND) symptoms and asymptomatic HLB. (Figure panels for ND and asymptomatic HLB were adapted from Sanchez *et al*., 2019, Anal. Bioanal. Chem.).

**Figure 2:**
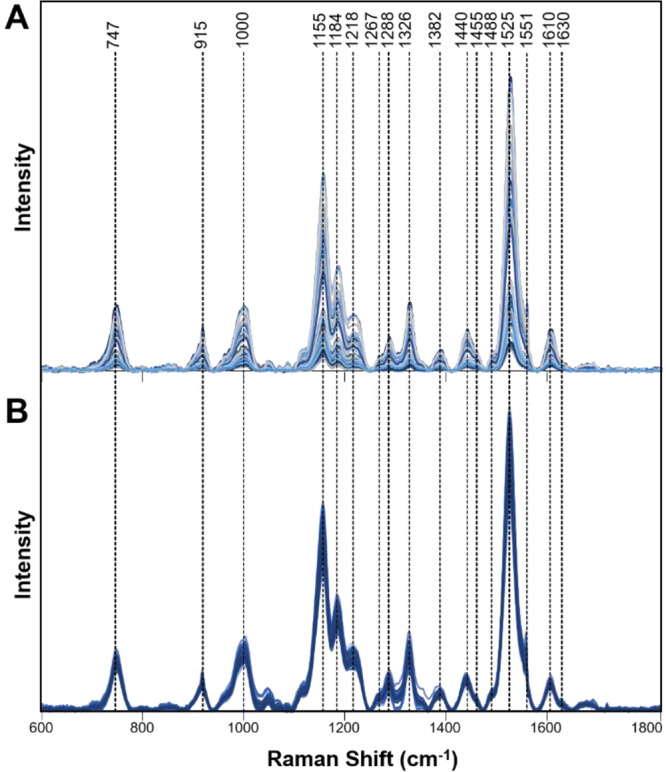
Raman spectra collected from leaves of IFH (**A**) and GHH (**B**) grapefruit trees.

**Figure 3:**
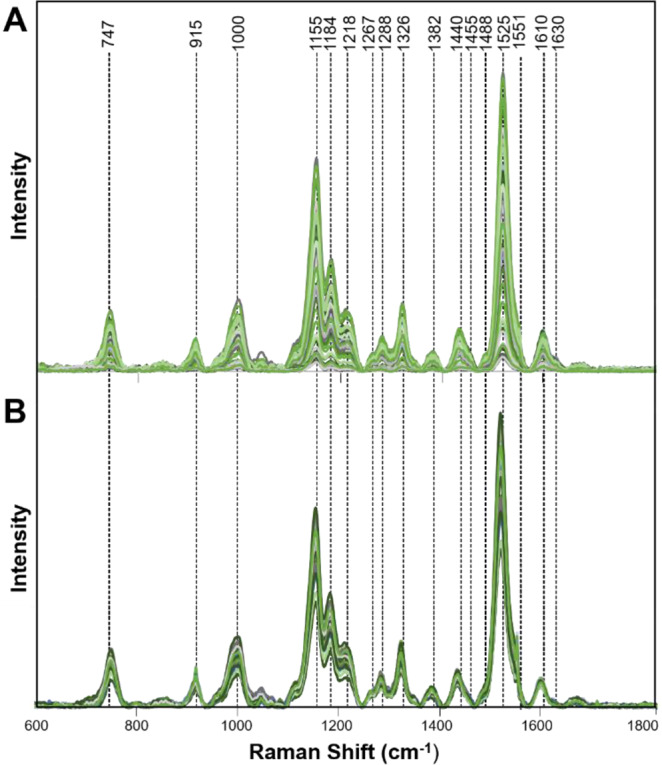
Raman spectra collected from leaves of IFH (**A**) and GHH (**B**) orange trees.

**Figure 4:**
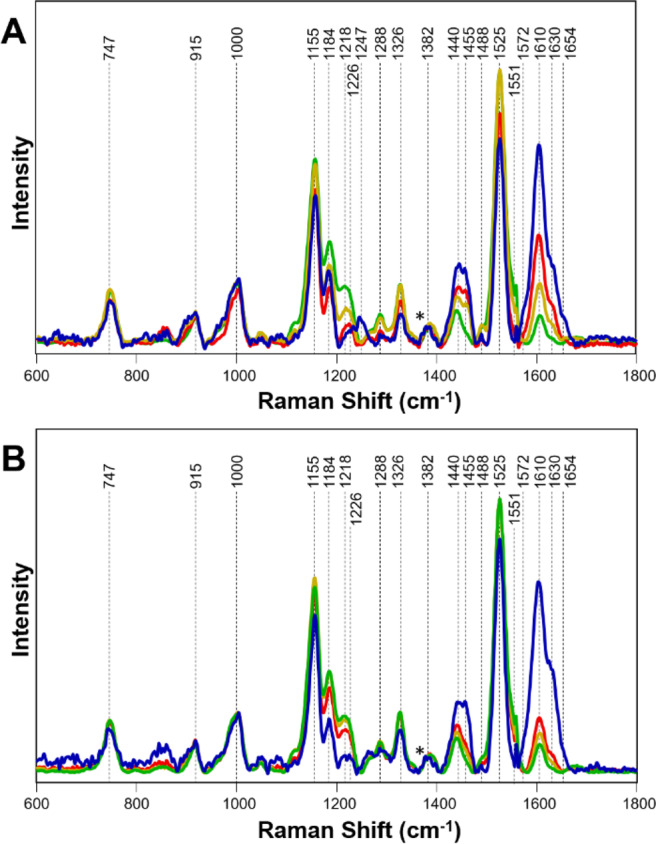
Raman spectra collected from leaves of GHH (green), IFH (gold), asymptomatic HLB infection (red), and nutrient-deficit (blue) symptoms in (**A**) grapefruit and (**B**) orange trees. Spectra are normalized on the CH_2_ vibrational band that is present in nearly all classes in biological molecules (marked by asterisks (*)).

To answer these questions, we collected Raman spectra from the leaves of healthy orange and grapefruit citrus trees grown in the greenhouse (GHH) with no insect exposure (Fig. 1). We compared these spectra to the spectra collected from IFH plants (Figs. 2 and 3). We found that Raman spectra collected from leaves of IFH orange and grapefruit trees exhibited large variations in intensities of all vibrational bands (Table 1). Specifically, we found significant changes in intensities of lignin/phenylpropanoids (1610 cm^−1^) and carotenoids (1000 cm^−1^, 1527–1551 cm^−1^) bands, as well as in intensities of CH_2_ and CH_3_ vibrations (1382–1488 cm^−1^). Such changes point out on possible presence of HLB or ND (Fig. 4)^18^. We showed that both HLB and ND on citrus trees were associated with an increase in the intensity of lignin/phenylpropanoids band (1610 cm^−1^), as well as a decrease in the intensity of the vibrational band at 1525 cm^−1^, which can be assigned to carotenoids. These changes indicate that both HLB and ND are associated with the increase in the lignin/phenylpropanoid content of the plant and degradation of its carotenoids. Both HLB and ND are also associated with a change in intensity of 1155 and 1184 cm^−1^ bands, suggesting that these plants can experience both HLB disease and ND. We also observed a decrease in the intensity of 1288 cm^−1^ and an increase in the intensity of 1440–1455 cm^−1^ that are associated with both HLB and ND. At the same time, ND and HLB can be distinguished spectroscopically by the presence of a vibrational band at 1247 cm^−1^, which is specific evidence for ND plants only. This band was not observed in the Raman spectra collected from IFH plants (Fig. 4).

**Table 1:** Vibrational bands and their assignments for healthy, HLB positive leaves and leaves from orange and grapefruit trees with nutrient deficits (ND).

Band	Vibrational mode	Assignment
747	γ(C-O-H) of COOH	pectin^41^
915	ν(C-O-C) in plane, symmetric	cellulose, lignin^42^
1000	ν_3_ (C-CH_3_ stretching) and phenylalanine	carotenoids, protein^43,44^
1155	asym ν(C-C) ring breathing	carbohydrates, cellulose^42^
1184	ν(C-O-H) next to aromatic ring + σ(CH)	xylan^45,46^
1218–1226	δ(C-C-H)	aliphatic^47^, xylan^45^
1247	C-O stretching (aromatic)	lignin^48^
1288	δ(C-C-H)	aliphatic^47^
1326	δCH_2_ bending vibration	cellulose, lignin^42^
1382	δCH_2_ bending vibration	aliphatic^47^
1440	δ(CH_2_) + δ(CH_3_)	aliphatic^47^
1455	δCH_2_ bending vibration	aliphatic^47^
1488	δ(CH_2_) + δ(CH_3_)	aliphatic^47^
1527–1551	-C=C- (in plane)	carotenoids^49,50^
1610	ν(C-C) aromatic ring + σ(CH)	phenylpropanoids, lignin^51,52^
1630	C = C-C (ring)	phenylpropanoid, lignin^51–53^

Observed spectral changes helped to uncover molecular mechanisms perturbed during HLB infection. Carotenoids (1000 cm^−1^, 1527–1551 cm^−1^) are a critical component of the light harvesting complexes and protect plants from photo-oxidation during photosynthesis. A decrease in carotenoid intensity may suggest that photosynthetic processes are disrupted upon HLB infection and thus correlates with the blotchy mottle/chlorosis symptoms typical for HLB. Additionally, the carotenoids could be metabolized to apocarotenoids, or other volatile signaling molecules that could function as a stress adaptation^22^. Lignin and related secondary metabolites not only act as physical barriers to invading plant pathogens but can also play a key role in plant defense signaling^23^. An increase in the lignin/phenylpropanoid content has been shown as a plant response to various abiotic stresses^24–27^. Therefore, an increase in the intensity of these molecules (1610–1630 cm^−1^) in HLB infected samples suggests that plants build up these molecules to combat the proliferation of bacteria within the tissues. Our findings concur with previous proteomic, transcriptomic and metabolomic studies which showed that HLB-infected tissues had upregulation in lignin/ phenylpropanoid pathways, while carotenoid pathways were downregulated^28,29^.

Although both carotenoids and lignin/phenylpropanoid bands could be used to monitor HLB progression, we found that changes in the intensity of 1610 cm^−1^ band were more prominent compared to changes in the intensities of carotenoid vibrations. This suggests that a change in the intensity at 1610 cm^−1^ can be used for more accurate assessment of the development of HLB in plant. Therefore, this band has been selected to track progression of this disease in citrus plants (discussed below).

**Figure 5:**
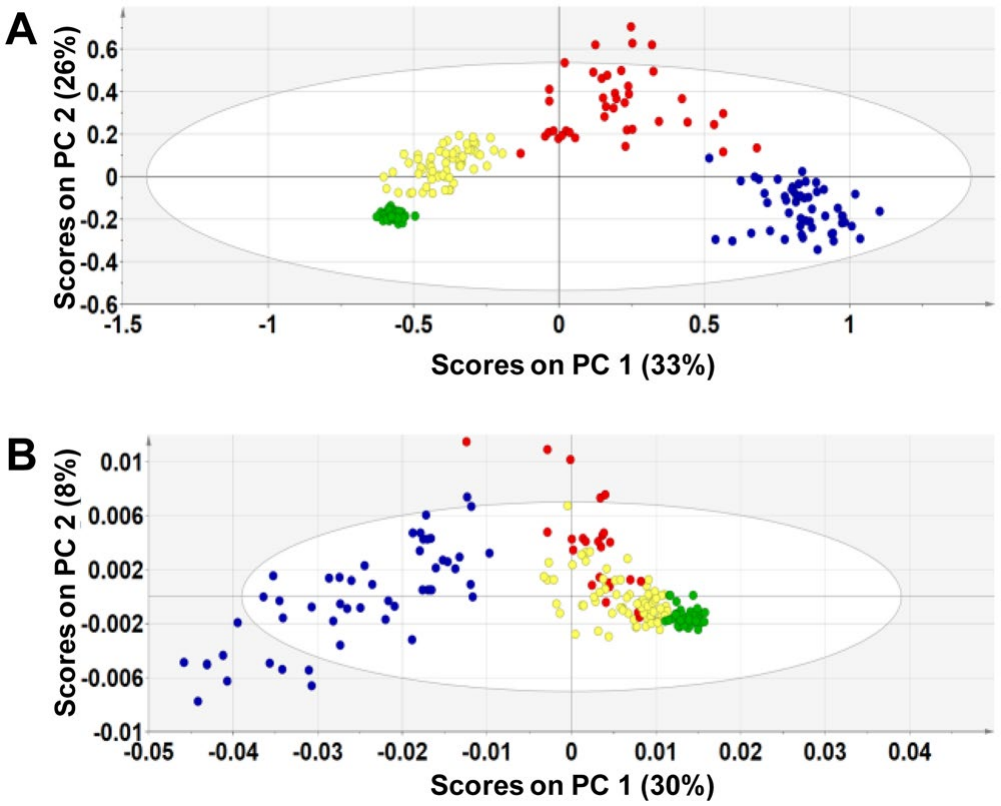
OPLS-DA loadings plot of Raman spectra collected from leaves of GHH (green), IFH (yellow), ND (blue) and early-stage HLB (red) grapefruit (**A**) and orange (**B**) trees.

In our previous work, we demonstrated that multivariate data analysis^30^ could be used to disentangle HLB and ND with high accuracy^18^. Using orthogonal partial least squares discriminant analysis (OPLS-DA), we analyzed IFH spectra collected from leaves of orange and grapefruit trees and compared them to the spectra acquired from GHH, ND and early-stage HLB trees (Fig. 5). Detailed analysis of OPLS-DA results is discussed in Supporting Information. We found that IFH orange and grapefruit spectra were localized in-between greenhouse healthy and asymptomatic HLB positive (PCR positive) spectral groups. At the same time, ND spectra were grouped separately aside from the IFH spectra. We also observed a partial overlap between IFH and HLB positive spectral groups that was small in the case of grapefruits and larger for oranges (Fig. 5). These changes in the intensities of vibrational bands in the spectra collected from IFH orange and grapefruit leaves, as well as the lack of homogeneity that can be observed for GHH spectra, suggests the possibility that the IFH trees could be trending towards becoming HLB positive. It should be noted that at the time of analysis, IFH were qPCR negative for HLB. However, CLas titers can accumulate overtime (discussed below) and possibly within 6–12 months from sampling, IFH trees could become HLB positive. To further clarify, the IFH group is described as ‘very early stage’ of HLB (no symptoms, qPCR negative), which is different from the ‘early stage’ group (no symptoms, qPCR positive). We also found that OPLS-DA enables highly accurate prediction of such very early stages of HLB infection (IFH), Tables 2 and 3. Specifically, 96.7% and 98.8% accuracy was achieved for IFH diagnostics for grapefruit and orange leaves, respectively. Taken together, our detailed analysis here of GHH, IFH, HLB-infected and ND citrus suggests that RS can be used for accurate diagnostics of HLB at a very early stage and that RS is at least as sensitive as qPCR, which has been the “gold standard” for diagnostics till date.

**Table 2:** OPLS-DA confusion matrix of GHH and IFH, as well as asymptomatic HLB-positive and ND classes of grapefruit leaves.

	Members	Correct	*Asymptomatic HLB-positive*	*IFH*	*GHH*	*ND*
*Asymptomatic HLB-positive*	40	92.5%	**37**	1	0	2
*IFH*	60	96.7%	0	**58**	2	0
*GHH*	48	100%	0	0	**48**	0
*ND*	52	100%	0	0	0	**52**
No class	43		33	3	1	6
Total	243	97.5%	70	62	51	60

**Table 3:** OPLS-DA confusion matrix of GHH and IFH, as well as asymptomatic HLB-positive and ND classes of orange leaves.

	Members	Correct	*Asymptomatic HLB-positive*	*IFH*	*GHH*	*ND*
*Asymptomatic HLB-positive*	24	54.17%	**13**	0	11	0
*IFH*	84	98.8%	0	**83**	1	0
*GHH*	48	97.9%	0	1	**47**	0
*ND*	51	98.0%	0	1	0	**50**
No class	43		33	3	1	6
Total	255	93.2%	46	88	60	56

**Figure 6:**
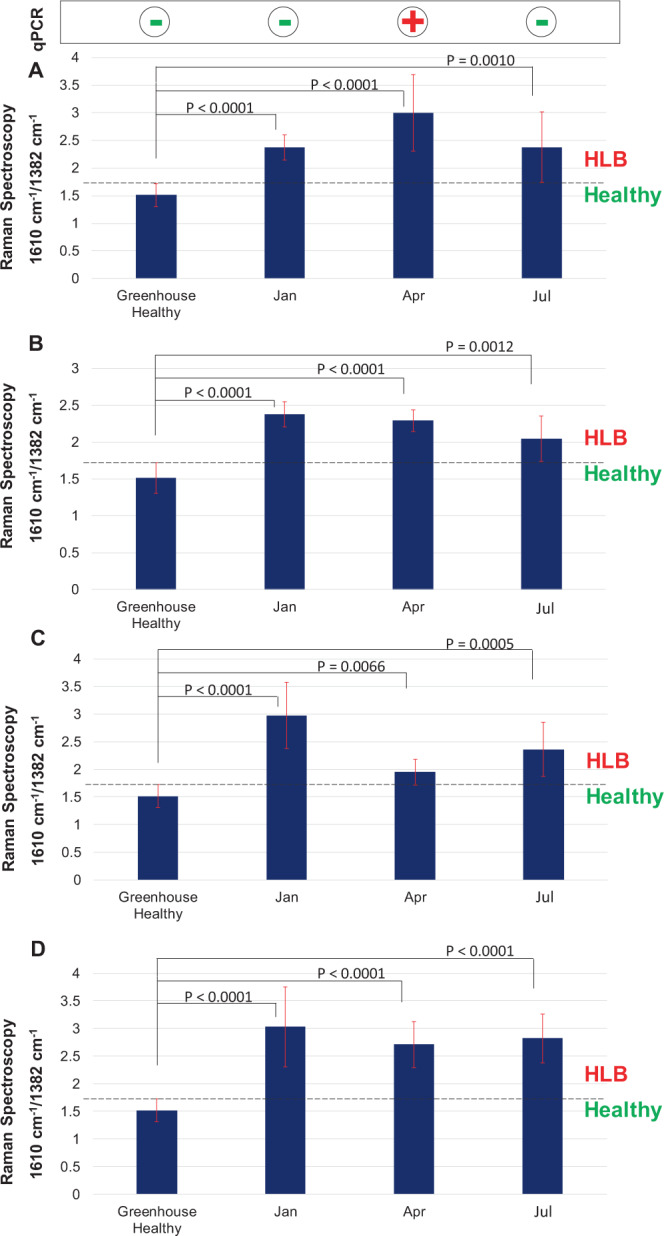
Histograms of intensity of 1610 cm^−1^ (lignin/phenylpropanoids) band normalized to the intensity of 1382 cm^−1^ band (CH_2_ vibrations) in the Raman spectra collected from four trees (**A**–**D**) over a 6 month period. The observed intensities are compared to the intensity of 1610 cm^−1^ band (with identical normalization) in the Raman spectra collected from GHH trees (greenhouse healthy). qPCR status of the plants is shown on the top panel. P-values obtained from two-sample t-tests are displayed between the compared groups.

To further evaluate this observation, and to compare diagnostic sensitivity between Raman and qPCR, we set up a temporal (time-course) sampling experiment for field trees. Four HLB negative orange trees were selected based on qPCR analysis in January, and were monitored for six months, taking readings every three months by RS and qPCR (Fig. 6). In the first round of Raman measurements, which were made in January, the intensity of the 1610 cm^−1^ band normalized on the intensity of 1382 cm^−1^ (CH_2_ vibrations) was in the range of 2.5–3.5, and was significantly higher than greenhouse healthy plants indicating it is possibly related to HLB. In the next monitoring period, when qPCR was done in April, all the four trees came HLB positive. However, the intensity of the 1610 cm^−1^ band normalized on the intensity of 1382 cm^−1^ in the Raman spectra collected in April was unchanged relative to the intensity of this band in the January spectra, and significantly higher compared to the intensity of this band in GHH plants.

This indicated that RS was able to detect HLB in the plants already in January, whereas qPCR could detect it only three months later (April), (Figures S3–S4). This experiment also allowed for quantitative measurement of the CLas/HLB threshold that can be used for highly accurate Raman-based diagnostics of HLB. We found that intensity of 1610 cm^−1^ band normalized on the intensity of 1382 cm^−1^ below 1.925 corresponds to healthy orange trees, whereas intensities above this value indicate presence of CLas/HLB.

Interestingly, during the final round of measurements, which were made in July, all four trees that were HLB positive in April appeared to be negative by qPCR (Figures S1–S4). Such qPCR negative results are typical for hot summer months, as the bacteria clears in the shoots due to high temperatures, which were in the range of 100–120 °F during May-August. Although well-documented, this problem substantially complicates qPCR-based diagnostics of HLB^31–34^. The Raman readings, however, reported stable signal intensity of 1610 cm^−1^ band above 1.925 (Fig. 6). These results demonstrate that RS reliably detects HLB-infected trees, regardless of temporal and spatial fluctuations of CLas in the HLB-infected trees.

In summary, our findings demonstrate RS can be used for confirmatory, non-invasive and non-destructive prediction of HLB much earlier than it can be diagnosed by qPCR. We also show that that the combination of sensitivity, specificity and the high throughput nature of RS technique makes it a valuable tool for plant pathology community^20,35,36^ and can specifically aid in timely intervention and management of the devastating HLB disease. Further development of the RS technology/analysis that will allow identification of minor structural differences of closely related chemicals should help identify additional signature peaks for very early stage HLB diagnostics, and to enhance the accuracy rates even more.

## Methods

### Plants material

Twelve leaf samples (three leaves from a branch per tree) were collected from four trees in greenhouse-grown healthy (GHH), in field-grown healthy (IFH), nutrient deficient (ND) and HLB early (non-symptomatic) of orange (*Citrus sinensis*, Valencia) and grapefruit (*Citrus paradasi*, Rio red) from Weslaco, Texas. Leaf samples were collected in Ziploc bags and immediately brought to laboratory for RS. After RS, the leaf samples were stored in −80 °C for DNA isolation. Four orange trees of IFH surrounded by *C*Las positive oranges trees were selected to monitor the growth of *C*Las over time. Three leaves were collected from the same branch of each tree to quantify *C*Las titer using qPCR and RS for every three months over 6 months, starting in January 2019.

### Raman spectroscopy

Raman spectra were taken with a hand-held Resolve Agilent spectrometer equipped with 830 nm laser source^18^. The following experimental parameters were used for all the collected spectra: 1 s acquisition time, 495 mW power, and baseline spectral subtraction by device software. Four spectra were collected from each leaf from four quadrants on the adaxial side of the leaf. For grapefruit, IFH had 60 samples, GHH had 48 samples, ND had 52 samples, and asymptomatic had 40 samples. For orange, IFH had 84 samples, GHH had 48 samples, ND had 51 samples, and asymptomatic had 24. Spectra shown in the manuscript are raw baseline corrected, without smoothing.

### DNA extraction

After taking Raman spectra readings^18^, DNA was extracted from each leaf, according to Alonso and Patricia (2014) with minor modifications as follow: ~200 mg of finely sliced leaf tissue was homogenized in 2 ml screw-cap microcentrifuge tubes for 45 s at 5000 rpm with the Precellys 24 homogenizer (MO BIO Laboratories, Carlsbad, CA, USA) in the presence of two steel BB air gun beads (BB refers to the bead size, 4.5 mm-diameter) (Walmart Supercenter, Bentonville, AR, USA). DNA was quantified by measuring the concentration using a NanoDrop 1000 Spectrophotometer (Thermo Fisher Scientific, Wilmington, DE, USA). The quality of DNA was examined by electrophoresis on 1% agarose gels stained with ethidium bromide.

### DNA isolation and quantitative real-time PCR (qPCR)

Leaf DNA was isolated according to Rezadoost and Chiong^37,38^ with minor modifications as follow: leaf samples were finely chopped, and ~200 mg of leaf tissues was taken in 2 ml screw-cap microcentrifuge tubes^18^. The samples were homogenized for 45 s at 5000 rpm with the Precellys 24 homogenizer (MO BIO Laboratories, Carlsbad, CA, USA) in the presence of two steel BB air gun beads (BB refers to the bead size, 4.5 mm-diameter) (Walmart Supercenter, Bentonville, AR, USA). Quantity and quality of DNA was measured by NanoDrop 1000 Spectrophotometer (Thermo Fisher Scientific, Wilmington, DE, USA) and electrophoresis on 1% agarose gel respectively.

Quantitative real-time PCR (qPCR) was performed using a CFX384 Real-Time PCR Detection System (Bio-Rad Laboratories, Inc., Hercules, CA, USA). The qPCR reaction mix consist of 0.4 µl of each forward and reverse primers, 5 µl of iTaq universal SYBR Green supermix (Bio-Rad Laboratories, Inc.), 1 µl of genomic DNA (50 ng), and 3.2 µl of nuclease free water. Forward primer RNR-F (5′-GGATAGTCCTGTTATTGCTCCTAAA-3′) and reverse primer RNR-R (5′-ACAAAAGCAGAAATAGCACGAACAA-3′) combination was used to amplify the gene encoding β-subunit of ribonucleotide reductase (*RNR)* of the *C*Las^39^. A citrus endogenous gene Glyceraldehyde-3-phosphate dehydrogenase C2 (GAPC2) was used as internal control for normalization of qPCR data. All reactions were performed in duplicate, including the non-template control reactions using the following conditions: one cycle at 95 °C for 3 min, 39 two-step cycles each at 95 °C for 15 s and 57 °C for 30 s, and a final melting curve of 65–95 °C for 5 s. The resulting Ct (threshold cycle) values were normalized with the citrus housekeeping gene GAPC2^40^. In our experimental condition, we selected cut off value of Ct ≤28 to differentiate heathy from HLB positive (Figures S1 and S2).

### Multivariate data analysis

SIMCA 14 (Umetrics, Umea, Sweden)^18^ was used for statistical analysis of the collected Raman spectra. All imported spectra were scaled to unit variance to give all spectral regions equal importance. Orthogonal partial least squares discriminant analysis (OPLS-DA) was performed in order to determine the number of significant components and identify spectral regions that best explain the separation between the classes. In order to give each of the spectral regions equal importance, all spectra were scaled to unit variance. Raw spectra, containing wavenumbers 350–2000 cm^−1^, were retained in the model that resulted from this iteration of OPLS-DA.

## Supplementary information

Supplementary Information.
