# Supplementary material for: Raman Spectroscopy vs Quantitative Polymerase Chain Reaction In Early Stage Huanglongbing Diagnostics
Source: Sci Rep. 2020 Jun 22;10:10101. doi: 10.1038/s41598-020-67148-6 (PMC7308309; doi:10.1038/s41598-020-67148-6)
Supplement: Supplementary file 1 — Supplementary Information. [file 41598_2020_67148_MOESM1_ESM.docx]

Raman Spectroscopy *vs* Quantitative Polymerase Chain Reaction In Early Stage Huanglongbing Diagnostics

Lee Sanchez^1^, Shankar Pant,^2¶^ Kranthi Mandadi^2,3^* and Dmitry Kurouski^1,4^*

1. Department of Biochemistry and Biophysics, Texas A&M University, College Station, Texas 77843, United States
2. Texas A&M AgriLife Research and Extension Center at Weslaco, Texas 78596, United States
3. Department of Plant Pathology and Microbiology, Texas A&M University, College Station, Texas 77843, United States
4. The Institute for Quantum Science and Engineering, Texas A&M University, College Station, Texas, 77843, United States

¶ Current address: Agricultural Research Service, U.S. Department of Agriculture, Stillwater, OK, United States

**Supporting Information**

***Grapefruit data analysis:*** The loading plot and misclassification table were then generated using this final model, which contained 3 predictive components, 1 orthogonal component, and 1536 (458-1993 cm^-1^) original wavenumbers for standard normal variate (SNV) pre-processed first derivative spectra. Predictive components (PC) one, two, and three explained 33%, 26%, and 19% of variation between the different classes, respectively. Absolute intensities in the loadings spectra were proportional to the percentage of the total variation between classes explained by each wavenumber within each component. The model identified the lignin peak at 1248 cm^-1^ (PC1), cellulose/lignin at 1325 cm^-1^ (PC1), aliphatic bands at 1456 cm^-1^ and 1485 cm^-1^ (PC1), carotenoids at 1537 cm^-1^ (PC1), carbohydrates/cellulose band at 1155 cm^-1^ (PC2) and lignin peak at 1603 cm^-1^ and 1630 cm^-1^ (PC1) as the strongest predictors of the pathogens, which supports the conclusions of our qualitative spectral analysis presented above. The model also explained 60% of the variation (R2X) in the spectra and 78% (R2Y) of the variation between the classes.

Loadings plots:


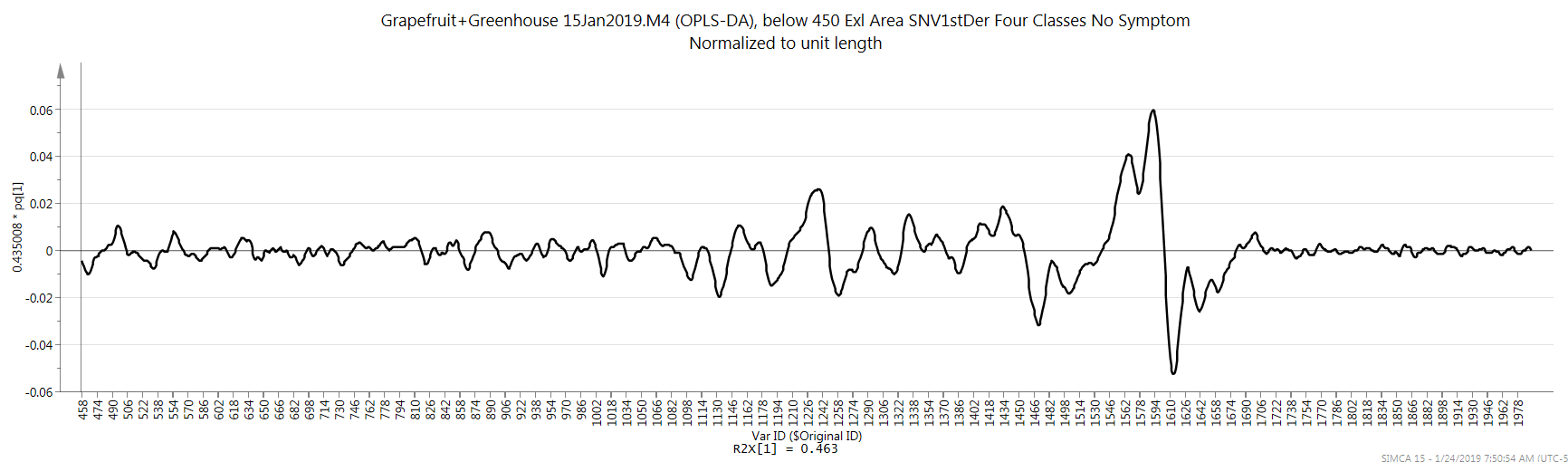


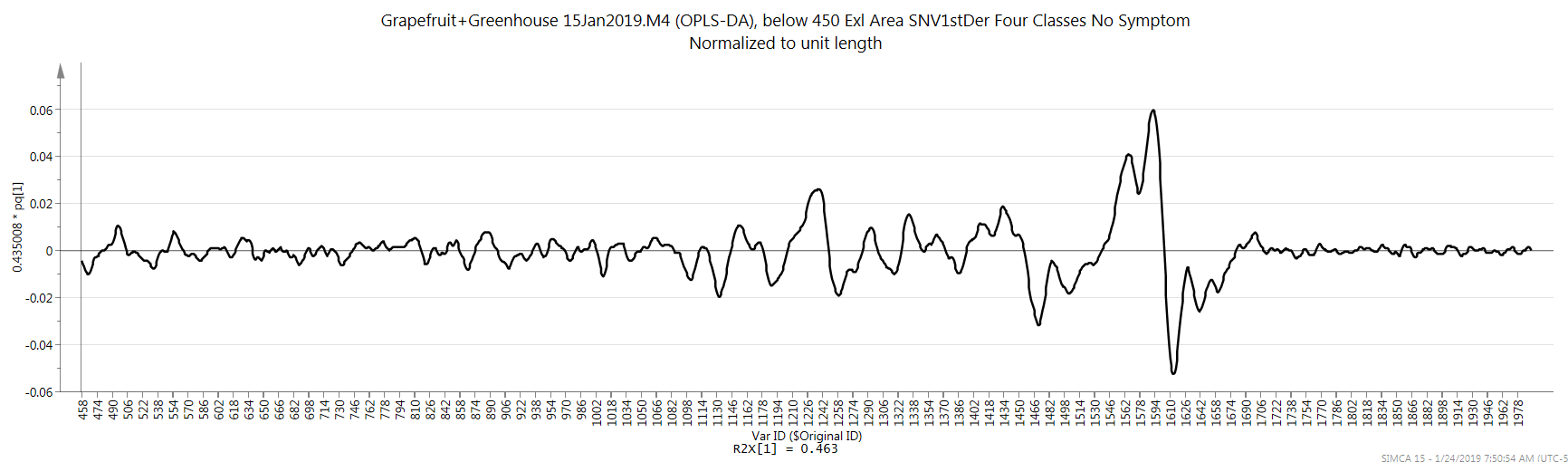


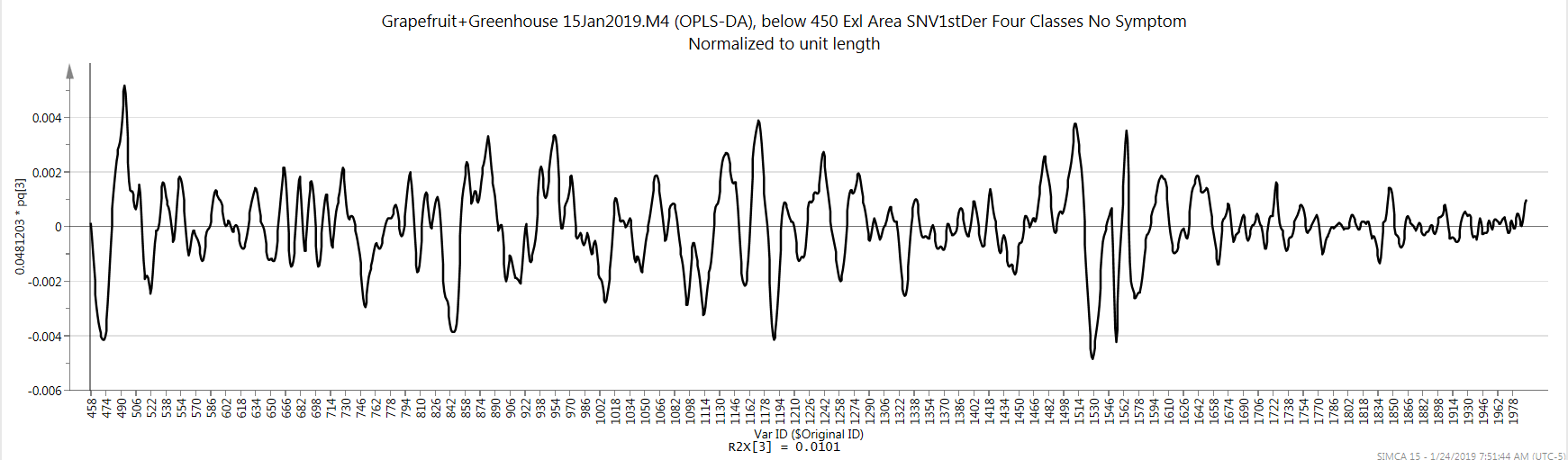


***Orange data analysis:*** The loading plot and misclassification table were then generated using this final model, which contained 3 predictive components, 1 orthogonal component, and 1550 (451-2000 cm^-1^) original wavenumbers for area-normalized spectra. Predictive components (PC) one, two, and three explained 30%, 8%, and 15% of variation between the different classes, respectively. Absolute intensities in the loadings spectra were proportional to the percentage of the total variation between classes explained by each wavenumber within each component. The model identified the pectin peak at 749 cm^-1^ (PC1), cellulose/lignin peaks at 915 cm^-1^ (PC1), carotenoids/protein peaks at 1000 cm^-1^ (PC1), the carbohydrates/cellulose band at 1155 cm^-1^ (PC1), carotenoids at 1527 cm^-1^ (PC1), as well as the lignin peak at 1603 cm^-1^ (PC1) as the strongest predictors of the pathogen, which supports the conclusions of our qualitative spectral analysis presented above. The model also explained 90% of the variation (R2X) in the spectra and 53% (R2Y) of the variation between the classes.

Loadings plots:


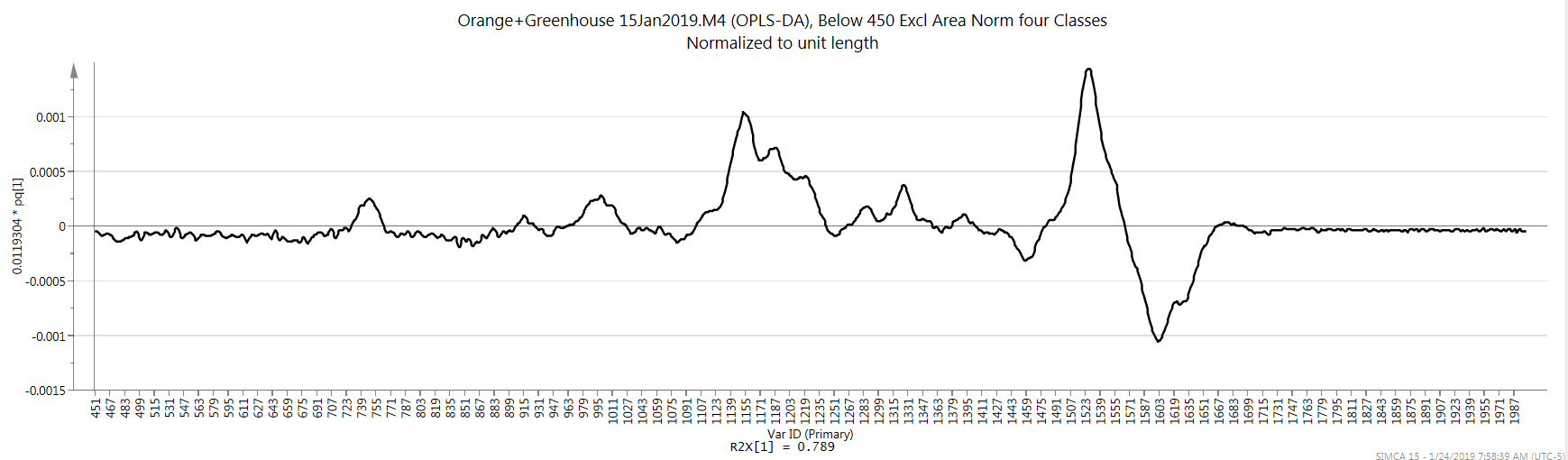


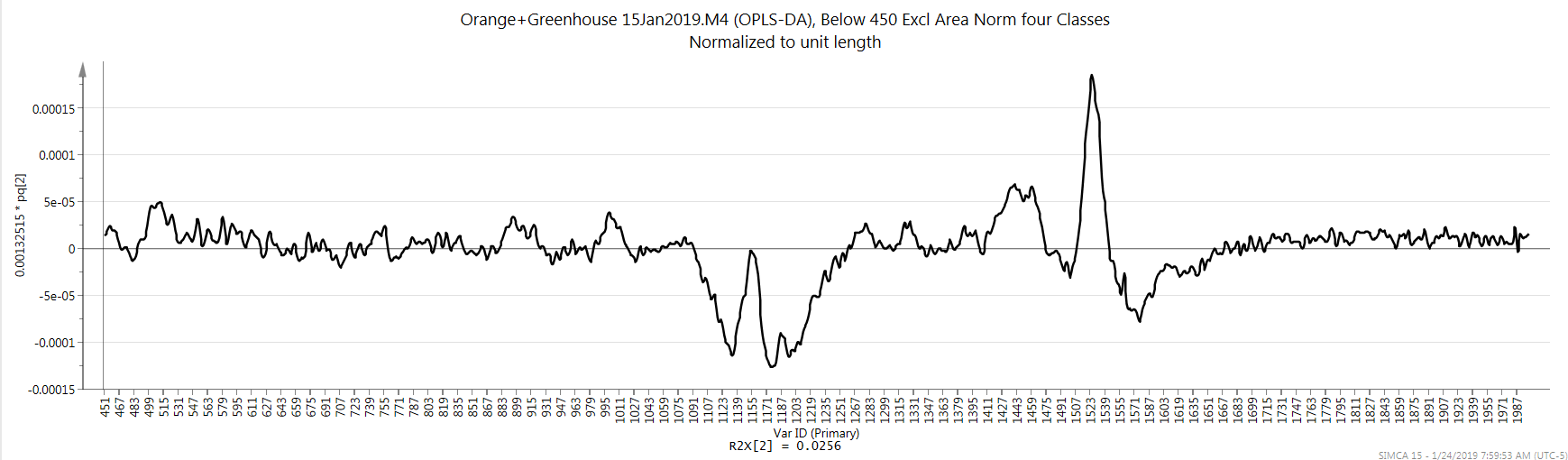


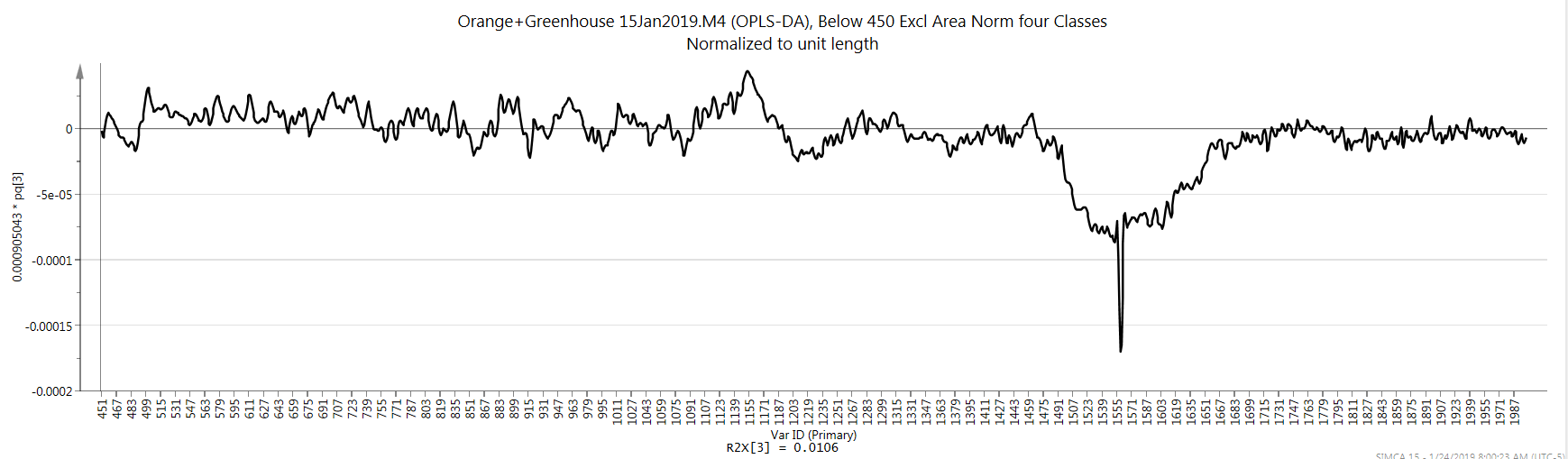


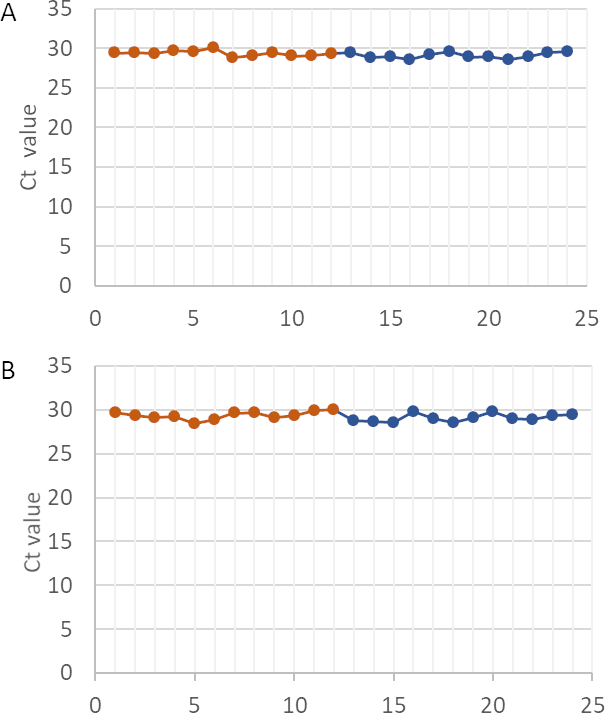


Figure S1. Quantitative real time PCR (qPCR)-based diagnostics of IFH (orange) and GHH (blue) leaf DNA samples from (A) orange and (B) grapefruit trees.


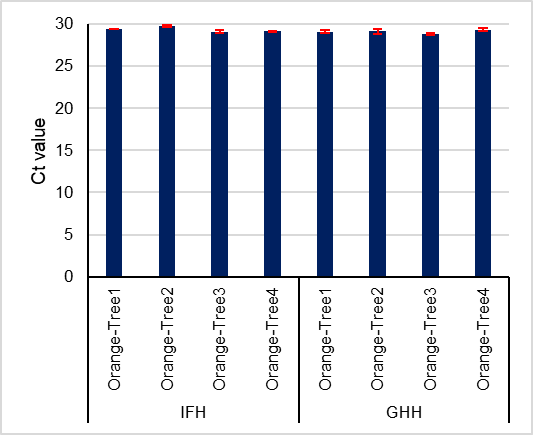

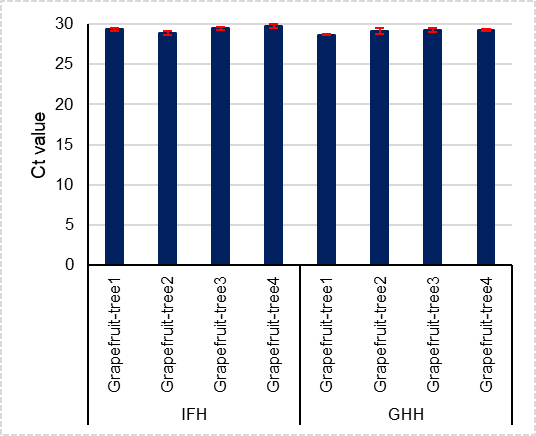


A

B

Figure S2. Quantitative real time PCR (qPCR)-based diagnostics of IFH and GHH leaf DNA samples from (A) orange and (B) grapefruit trees. Dashed black line is the normalized Ct cutoff value (Ct ≤ 28) for HLB-positive.


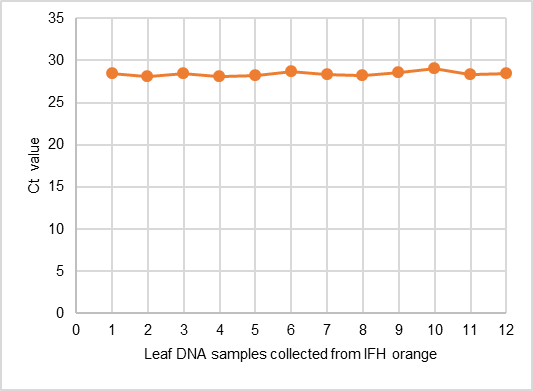


A


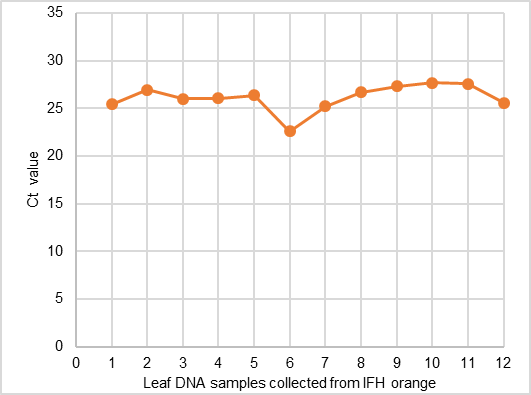


B


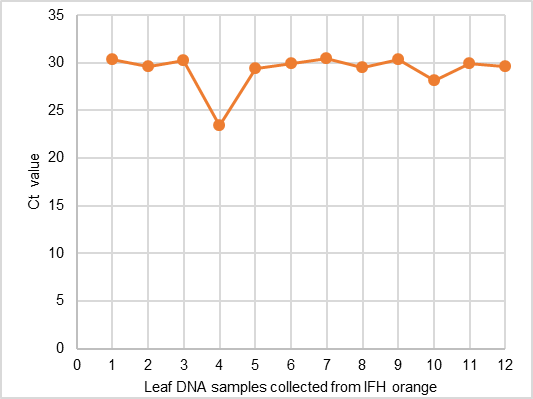


C

Figures S3. Quantitative real-time PCR (qPCR)–based monitoring of IFH orange leaf trees over six months. Leaf samples collected in (*A*) January, (*B*) April, (*C*) July. Solid black line is the normalized Ct cutoff value (Ct ≤ 28) for HLB-positive (below).


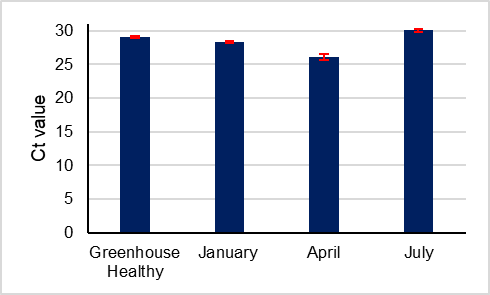

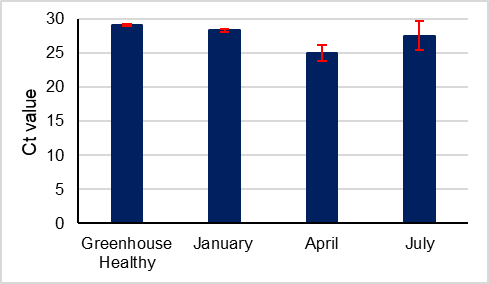

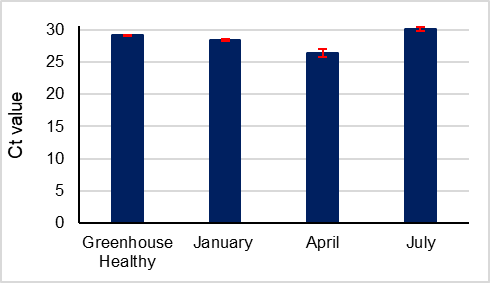

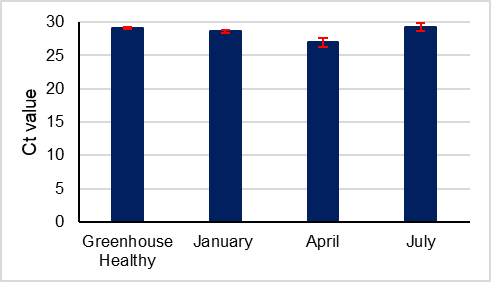


A

B

C

D

Figures S4. Histograms of Ct value obtained from qPCR analysis in leaf genomic DNA collected from (*A-D*) four trees during 6 months’ period. Dashed black line is the normalized Ct cutoff value (Ct ≤ 28) for HLB-positive.
